# LncRNA-CFTBS enhances *Mycobacterium tuberculosis* survival in macrophages by modulating ferroptosis through the miR-515-5p/miR-519e-5p/SAT1 axis

**DOI:** 10.1080/21505594.2025.2545563

**Published:** 2025-10-11

**Authors:** Yuxin Li, Yajuan Bai, Chunyu Hei, LiYing Zhao, Wenqi Dong, Chen Tan

**Affiliations:** a National Key Laboratory of Agricultural Microbiology, College of Veterinary Medicine, Huazhong Agricultural University, Wuhan, China; b College of Fisheries, Huazhong Agricultural University, Wuhan, China; c Hubei Hongshan Laboratory, Huazhong Agricultural University, Wuhan, China; d Key Laboratory of Preventive Veterinary Medicine in Hubei Province, Huazhong Agricultural University, Wuhan, China

**Keywords:** Mycobacterium tuberculosis, long noncoding RNAs, miRNA, ferroptosis, SAT1

## Abstract

Tuberculosis (TB) remains one of the primary global causes of death and poses substantial public health challenges. The intracellular survival of *Mycobacterium tuberculosis* (M.tb) can be influenced by ferroptosis; however, how lipid peroxidation-induced ferroptosis operates during M.tb infection remains unclear. Our study revealed a significantly upregulated lncRNA (lncRNA-cytoplasm-regulating ferroptosis and tuberculosis survival (CFTBS)) that modulates ferroptosis, enhancing M.tb intracellular survival by affecting the lipid peroxidation-related pathway rather than the cystine/GSH/GPX4 pathway. We elucidated that lncRNA-CFTBS competitively binds miR-515-5p and miR-519e-5p, regulating spermidine/spermine N1-acetyltransferase 1 (SAT1) expression, which plays a critical role in increasing the expression of arachidonic acid 15-lipoxygenase (ALOX15) and promoting lipid peroxidation and ferroptosis. Our findings reveal a mechanism by which lncRNA-CFTBS enhances M.tb survival during infection by regulating a noncanonical ferroptosis signalling pathway, offering a deeper understanding of the function of noncoding RNAs in ferroptosis and TB pathogenesis and identifying potential therapeutic targets.

## Introduction

The World Health Organization (WHO) reported that tuberculosis (TB) ranks as the second most lethal infectious disease, following COVID-19, with a mortality rate nearly double that of HIV [1]. A comprehensive understanding of the molecular mechanisms underlying *Mycobacterium tuberculosis* (M.tb) infection and intracellular persistence is crucial for overcoming the challenges associated with TB prevention and developing effective therapeutic strategies to improve treatment outcomes [2–4].

Ferroptosis is a nonapoptotic process characterized by the toxic accumulation of intracellular lipid peroxides driven by ferrous iron [5]. Several pathways influence ferroptosis [6,7], including the cystine/glutathione (GSH)/glutathione peroxidase 4 (GPX4) axis [8], the spermidine/spermine N1-acetyltransferase 1 (SAT1) pathway [9], and other mechanisms such as iron metabolism dysregulation [10], p53 regulation [11], and the FSP1‒CoQ10 axis [12]. The GSH‒GPX4 pathway functions as a major antioxidant system [13]. The SAT1 pathway is involved in polyamine catabolism, resulting in the generation of ROS and lipid peroxidation [14]. Arachidonic acid 15-lipoxygenase (ALOX15) acts downstream of SAT1, further contributing to oxidative lipid damage and ferroptosis [15,16]. Several studies indicate a link between ferroptosis and M.tb pathogenesis [17–19], and research on ferroptosis regulated by long noncoding RNAs (lncRNAs) in the context of M.tb is scarce. Existing studies on M.tb and ferroptosis have focused primarily on the cystine/GSH/GPX4 axis. For example, research has shown that during M.tb infection, the expression of GPX4 is downregulated [20], and studies using GPX4-deficient mice have demonstrated significantly increased lung necrosis and bacterial burdens, highlighting the protective role of GPX4 against M.tb-induced ferroptosis [21]. However, exploration of the non-GPX4 pathway in the iron death pathway in the M.tb infection process is still very limited.

LncRNAs play essential roles in gene regulation, influencing chromatin modification, transcriptional control, and posttranscriptional processing [22–24]. The subcellular localization of lncRNAs often determines their functional roles, with cytoplasmic lncRNAs frequently acting as molecular sponges for miRNAs [25–27]. By sequestering miRNAs, these lncRNAs can modulate miRNA target gene expression, thereby influencing various cellular pathways [28–30]. Moreover, lncRNAs control gene expression both transcriptionally and post-transcriptionally, further underscoring their versatile roles in cellular functions [31,32]. Recent research has underscored the crucial roles of ncRNAs in regulating various cellular processes during pathogen infections [33–35]. Extensive research has shown that lncRNAs are involved in ferroptosis. For instance, lncRNA PVT1 has been shown to inhibit ferroptosis signalling pathways by sequestering miR-195-5p [36]. In a hypoxic mouse model, knockout of the lncRNA hz06 in mice effectively inhibited ferroptosis [37]. Additionally, the lncRNA RGMB-AS1 stimulates ferroptosis by interacting with the RGMB-AS1-HMOX1 and RGMB-AS1-NAA10 axes [38]. The intricate mechanisms by which lncRNAs may influence ferroptosis during M.tb infection remain largely unexplored, indicating a significant gap in our understanding of the interplay between lncRNA-mediated regulatory pathways and M.tb-induced ferroptosis. Further investigations are needed to elucidate these potential interactions and their implications for tuberculosis pathogenesis and treatment.

This study focused on clarifying the effect of lncRNA-cytoplasm-regulating ferroptosis and tuberculosis survival (CFTBS)) on M.tb survival within macrophages and its underlying mechanisms. Our findings indicate that the ferroptosis pathway regulated by lncRNA-CFTBS, which diverges from the conventional cystine/GSH/GPX4 axis, predominantly involves lipid and iron metabolism pathways, wherein lncRNA-CFTBS competitively binds miR-515-5p and miR-519e-5p, thereby modulating the expression of their target gene, SAT1. Our study provides novel insights into the regulatory networks involving lncRNAs and miRNAs in M.tb infection, underscoring their potential as viable therapeutic targets for TB treatment.

**Figure:**
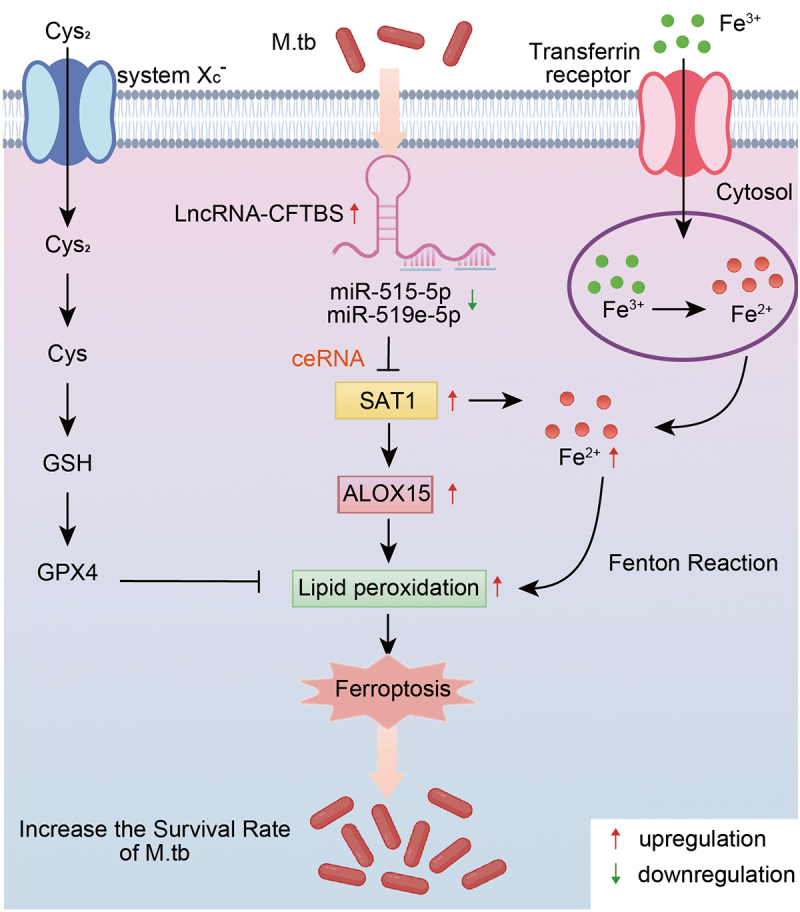

## Materials and methods

### Cell culture and bacterial strains

THP-1 macrophages (ATCC TIB-202) and HEK-293T cells (ATCC CRL-3216) were preserved in our laboratory, where they were expanded and passaged for further use. RPMI-1640 with 10% FBS was used to culture THP-1 macrophages, while HEK293T cells were grown in DMEM supplemented with 10% FBS at 37°C with 5% CO_2_.

Middlebrook 7H9 medium (Becton Dickinson, Difco 271,310), supplemented with 10% OADC, 0.5% glycerol (Sigma Aldrich, G5516), and 0.05% Tween 80 (Sigma Aldrich, P8074), was used to culture the M.tb H37Rv (ATCC 27,294) and BCG Tokyo (ATCC 35,737) strains.

### Macrophage infection

THP-1 cells were treated with PMA for 48 hours to induce macrophage differentiation, followed by infection with the M.tb H37Rv strain or BCG strain. After 4 hours, PBS washes were used to eliminate extracellular bacteria, and the cells were maintained in RPMI 1640 with 2% FBS at 37°C.

Samples for bacterial counts, RNA extraction, or protein extraction were collected at 4, 12, 24, and 48 hours postinfection.

### Colony-forming unit (CFU) assay

To assess bacterial survival within cells, infected THP-1 cells were lysed with 0.025% Triton X-100. The resulting lysates were serially diluted and plated on Middlebrook 7H11 agar supplemented with 10% OADC. Colony counts were conducted after a 3-week incubation at 37°C.

After BCG infection of THP-1 and lncRNA-CFTBS-overexpressing cells, different pathway inhibitors were used: DMSO (NC; Sigma, D8418), the autophagy inhibitor CQ (MCE, HY-17589A), the necrosis inhibitor Nec-1 (MCE, HY-15760), and the apoptosis inhibitor Z-VAD (Beyotime, C1202). The BCG MOI was set at 20, and CFU counts were conducted 24 hours postinfection.

### RNA fluorescence in situ hybridization (FISH)

LncRNA-CFTBS subcellular localization was determined by RNA FISH. THP-1 cells were fixed in 4% paraformaldehyde and then permeabilized with 0.1% Triton X-100 before hybridization with a fluorescently labelled probe specific to lncRNA-CFTBS. Nuclei were stained with DAPI, and imaging was performed using a laser scanning confocal microscope.

### Subcellular fractionation

Nuclei and cytoplasm isolation from THP-1 cells was performed using a PARIS^TM^ Kit (Thermo Fisher Scientific, AM1921) according to the provided protocol. THP-1 cells were washed with PBS, and incubated in 300 μL of cold cell fractionation buffer. After 3 minutes of centrifugation, the supernatant containing the cytoplasmic extract was transferred to a new RNase-free tube, and the pellet was washed with PBS and retained as the nuclear fraction.

### RNA isolation and quantitative real‒time PCR (qRT‒PCR)

Cells were lysed to extract total RNA using TRIzol reagent (Invitrogen, 15596026CN) as per the provided protocol. The extracted RNA was reverse transcribed into cDNA with the reverse transcription reagent (Vazyme, R233, China). Target gene expression was quantified using a quantitative PCR detection kit (Vazyme, Q111). For miRNA analysis, the tailing method was employed, followed by reverse transcription using the miRcute Plus First-Strand cDNA Kit (Tiangen, KR21). Quantitative analysis was subsequently conducted using a qPCR kit with SYBR Green detection (Tiangen, FP411). The primer sequences and corresponding analytical methods are provided in Table S1 of the supplementary material.

### Dual-luciferase reporter assay

The SAT1 3’-UTR and lncRNA-CFTBS sequences, including miR-515-5p/miR-519e-5p binding site sequences, were inserted into the pmirGLO vector, generating the SAT1-WT, SAT1-Mut, lncRNA-CFTBS-WT, and lncRNA-CFTBS-Mut constructs. HEK-293T cells were transfected with the constructs, miRNA mimics or controls. After 24 hours, an assay kit (Vazyme, DL101–01) was used to measure the luciferase activity, and their ratio was calculated.

### Western blotting

Cells were lysed in RIPA buffer (Beyotime, P0013B), followed by centrifugation. The supernatants were combined with SDS loading buffer and boiled. Proteins were separated via 15% SDS‒PAGE and transferred to membranes (Millipore, ISEQ00010). The membranes were blocked and then incubated with primary antibodies. After the membranes were washed, secondary antibodies were applied. Protein levels were quantified using a gel imaging system (Bio-Rad, #1705060). β-actin was used as the loading control. Anti-β-actin mouse mAb (Cell Signaling Technology, 8H10D10), HRP-conjugated goat anti-rabbit IgG (ABclonal, AS014), HRP-conjugated goat anti-mouse IgG (ABclonal, AS003), anti-SAT1 (ABclonal, A2506), anti-ferritin heavy chain (ABclonal, A19544), anti-ALOX15 (ABclonal, A6864), anti-SLC7A11/xCT (ABclonal, A2413), and anti-GPX4 (ABclonal, A25009) antibodies were diluted 1:2000 in blocking solution.

### Determination of the Fe^2+^ content

The measurement of Fe^2+^ levels was carried out using an intracellular Fe^2+^ content kit (Solarbio, BC5415) following the manufacturer’s protocol after exposing THP-1 cells to BCG for 24 hours. After infection, the appropriate amount of Reagent 1 was added on the basis of the number of cells. After sonication on ice, the supernatant was collected and kept on ice for analysis. Standard solution, Reagent 2, and samples were added to the mixture. The supernatant was thoroughly mixed and transferred to a 96-well plate, after which the absorbance was measured at 593 nm. The Fe^2+^ content was quantified against a standard curve.

### Measurement of malondialdehyde (MDA) and ROS levels

MDA levels were measured using an MDA assay kit (Beyotime, S0131S). The cells were infected with BCG for 48 hours prior to the assay. Briefly, for each assay, 0.1 ml of lysis buffer or homogenate was used per million cells. After homogenization or lysis, the samples were centrifuged, and the supernatant was collected. For the standard curve, 0.1 ml of standard solutions of varying concentrations was added to centrifuge tubes, along with 0.1 ml of each sample for measurement. The samples were then treated with 200 μl of MDA detection solution, mixed thoroughly, and heated to 100°C. After centrifugation, the supernatant was transferred to a 96-well plate, after which the absorbance was measured at 532 nm.

ROS detection was performed according to the protocol provided with the ROS detection kit (Beyotime, S0033S). DCFH-DA was diluted to a 10 μM solution, and the cells were incubated with it. Following incubation, the cells were washed to remove any remaining extracellular DCFH-DA. Flow cytometric analysis was performed.

### Transmission electron microscopy (TEM) analysis

THP-1 cells were fixed in 2.5% glutaraldehyde with 0.1 M phosphate buffer and then postfixed with 1% osmium tetroxide. The cells were dehydrated, embedded, and ultrathin sections were prepared. These sections were stained before being examined under a transmission electron microscope. Arrows indicate damaged mitochondria (swelling, membrane rupture, cristae loss).

### Statistical analysis

Statistical analysis was performed using GraphPad Prism software. The data are expressed as means ± SD. Student’s t test was used for comparisons between two groups, whereas one-way ANOVA with Dunnett’s correction was applied for multiple group comparisons against a control. Two-way ANOVA was employed to analyse the effects of two independent factors and their interaction on the dependent variable, providing a more comprehensive evaluation of the data. Statistical significance is indicated as *p < 0.05, **p < 0.01, and ***p < 0.005; ns denotes nonsignificance.

## Results

### LncRNA-CFTBS enhances BCG survival in macrophages

**Figure 1.**
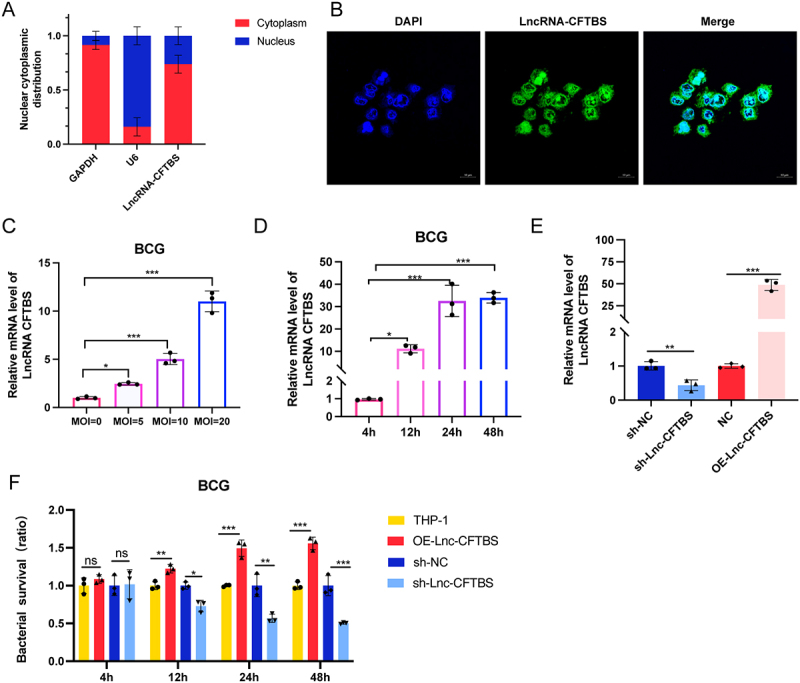
LncRNA-CFTBS expression and its impact on BCG survival in THP-1 cells.

In a previous study, we performed whole transcriptome sequencing of macrophages infected with M.tb [39]. That analysis revealed that the intergenic lncRNA ENST00000624048, which we have named lncRNA-CFTBS, is 1740 bp in length and is located on chromosome 12. LncRNA-CFTBS expression was notably upregulated in THP-1 cells after H37Rv infection, which was consistent with the sequencing data (Figure S1A and S1B). The localization of this lncRNA, initially confirmed by PCR following the separation of cytoplasmic and nuclear RNA (Figure 1(A)), was further substantiated by RNA FISH, which revealed that the green-labelled lncRNA-CFTBS was predominantly distributed in the cytoplasm (Figure 1(B)). We subsequently confirmed that the expression of lncRNA-CFTBS continuously increased with increasing MOI and time after BCG infection in THP-1 cells (Figure 1(C,D)). To explore the function of lncRNA-CFTBS, we constructed cell lines overexpressing lncRNA-CFTBS (OE-Lnc-CFTBS) and with lncRNA-CFTBS silencing (sh-Lnc-CFTBS), and validation of these cell lines confirmed successful overexpression and silencing of lncRNA-CFTBS at both the mRNA levels (Figure 1(E)). To explore whether lncRNA-CFTBS can affect the survival of BCG in macrophages, we assessed BCG CFU counts in cells expressing OE-Lnc-CFTBS or sh-Lnc-CFTBS at an MOI of 20. The results showed that OE-Lnc-CFTBS promoted the BCG CFU count in THP-1 cells, whereas sh-Lnc-CFTBS decreased the BCG CFU count (Figure 1(F)). In particular, 24 and 48 hours after infection, OE-Lnc-CFTBS increased BCG survival by approximately 1.5-fold compared with that of control cells. Conversely, sh-Lnc-CFTBS significantly reduced BCG survival, with a notable reduction of approximately 50% in BCG survival at 48 hours post infection. Additionally, we validated the function of lncRNA-CFTBS using transient transfection to silence lncRNA-CFTBS. Transfecting THP-1 cells with different lncRNA-CFTBS siRNAs and assessing the level of lncRNA-CFTBS via RT‒qPCR revealed that si-lncRNA2 had the best silencing effect; therefore, we used si-lncRNA2 for the subsequent experiments (Figure S1C). After transfecting THP-1 cells with lncRNA-CFTBS siRNA and subsequent BCG infection, we measured BCG survival at various time points. The results indicated that at 48 hours postinfection, the lncRNA-CFTBS siRNA-treated group presented an intracellular survival rate nearly half that of the siNC group (Figure S1D). The results demonstrated that lncRNA-CFTBS, which is located in the cytoplasm, is highly expressed in cells infected with M.tb, significantly influencing BCG intracellular survival.

(A) Nuclear and cytoplasmic distribution of lncRNA-CFTBS in THP-1 cells. (B) FISH analysis of lncRNA-CFTBS localization in THP-1 cells. LncRNA-CFTBS (green) is predominantly located in the cytoplasm (scale bar: 10 μm). (C-E) Relative mRNA levels of lncRNA-CFTBS in BCG-infected THP-1 cells across various MOIs, at various time points, and in sh-Lnc-CFTBS and OE-Lnc-CFTBS cell lines. (F) Impact of OE-LncRNA-CFTBS and sh-Lnc-CFTBS on BCG survival. The data are shown as means ± SDs. *p < 0.05, **p < 0.01, ***p < 0.005, and ns = not significant.

### LncRNA-CFTBS mediates ferroptosis in M.tb infection

**Figure 2.**
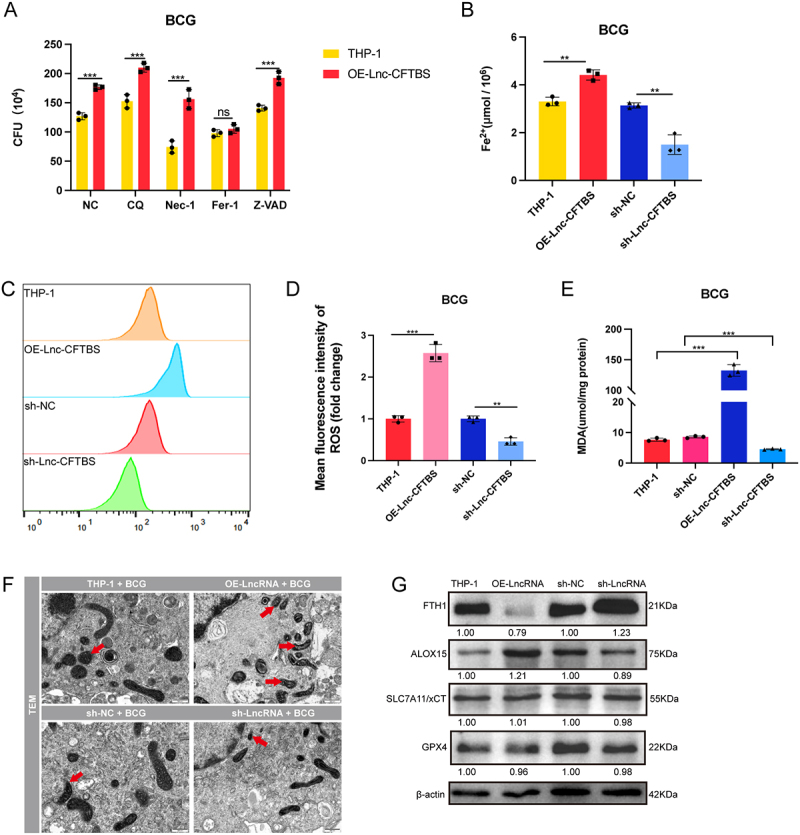
LncRNA-CFTBS regulates ferroptosis.

To investigate the pathways through which lncRNA-CFTBS influences the survival of BCG within the cells, THP-1 and OE-Lnc-CFTBS cells were exposed to BCG and subsequently treated with various pathway inhibitors, including an autophagy inhibitor (CQ), an apoptosis inhibitor (Z-VAD), a necrosis inhibitor (Nec-1), and a ferroptosis inhibitor (Fer-1),with a DMSO-treated group included as the control (NC). CFU counts were conducted 24 hours postinfection to determine bacterial survival. The results showed that the inhibition of the autophagy, necrosis, and apoptosis pathways did not alter the effect of lncRNA-CFTBS on BCG survival. However, the ferroptosis inhibitor Fer-1 eliminated the differences in BCG survival between THP-1 cells and OE-Lnc-CFTBS cells. These results indicate that lncRNA-CFTBS may exert its effects via modulation of the ferroptosis signalling pathway (Figure 2(A)).

Ferroptosis is a type of cell death marked by iron-dependent phospholipid peroxidation and the accumulation of reactive oxygen species (ROS), resulting in damage to the plasma membrane [40]. Additionally, ferroptosis increases ROS and MDA levels. Previous research has demonstrated that M.tb infection can trigger ferroptosis in macrophages [41,42], increasing the intracellular survival of bacteria. To investigate this phenomenon, we measured key indicators of ferroptosis. BCG infection of THP-1 cells resulted in elevated MDA levels, which were effectively suppressed by the ferroptosis inhibitor Fer-1. This observation suggests that BCG infection induces ferroptosis in THP-1 cells (Figure S2A). Furthermore, TEM images of BCG-infected THP-1 cells revealed abnormal mitochondrial morphology characteristic of ferroptosis. Treatment with Fer-1 ameliorated these changes (Figure S2B). These findings align with previous studies that have shown that ferroptosis enhances bacterial survival within host cells.

Ferroptosis is driven by the accumulation of ferrous ions, which catalyse the Fenton reaction, leading to increased ROS production, lipid peroxidation, and mitochondrial morphological changes [5,43,44]. To confirm the link between lncRNA-CFTBS and ferroptosis signalling during M.tb infection, we measured a series of ferroptosis-associated markers, including ferrous ion concentration, ROS levels, and MDA accumulation. We measured the intracellular Fe^2+^ levels in OE-Lnc-CFTBS and sh-Lnc-CFTBS cells following 24 hours of BCG infection. The results revealed that the level of intracellular Fe^2+^, which is positively correlated with ferroptosis, was significantly greater in OE-Lnc-CFTBS cells than in THP-1 cells. Conversely, Fe^2+^ levels decreased in sh-Lnc-CFTBS cells compared to sh-NC cells (Figure 2(B)). We used DCFH-DA probes to assess the impact of lncRNA-CFTBS on ROS levels during BCG infection through flow cytometry analysis. Flow cytometry analysis of ROS levels showed that, after BCG infection, OE-Lnc-CFTBS increased the mean fluorescence intensity of ROS, whereas sh-Lnc-CFTBS reduced it, indicating that lncRNA-CFTBS modulates ROS levels, contributing to ferroptosis (Figure 2(C,D)). MDA, a byproduct of lipid peroxidation and an indicator of ferroptosis, was assessed post-BCG infection. The results revealed a marked increase in MDA levels in OE-Lnc-CFTBS cells, whereas a decrease was observed in sh-Lnc-CFTBS cells, indicating that lncRNA-CFTBS overexpression promotes lipid peroxidation, whereas its silencing inhibits this process (Figure 2(E)). TEM provided further insight into the mitochondrial changes associated with ferroptosis. TEM images indicated that in THP-1 cells, OE-Lnc-CFTBS led to smaller mitochondria with disrupted and disordered cristae, indicative of more severe ferroptosis (Figure 2(F)). Counting of mitochondria with morphological abnormalities revealed an increase in the proportion of damaged mitochondria from 31.25% in the THP-1 BCG group to 74.29% in the OE-LncRNA BCG group. In contrast, lncRNA knockdown reduced this proportion from 25.71% in the shNC BCG group to 12.50% in the sh-LncRNA BCG group. Ferroptosis can occur through several pathways, primarily involving iron metabolism, lipid peroxidation, and the antioxidant system, with key pathways including the cystine-GSH-GPX4 pathway, iron metabolism, and lipid peroxidation [45,46]. The cystine-GSH-GPX4 axis is a major antioxidant defence mechanism, where SLC7A11 mediates cystine uptake and GPX4 reduces lipid hydroperoxides [47]. Iron metabolism pathways involve proteins such as ferritin heavy chain 1 (FTH1), which stores iron and regulates iron availability [48]. Lipid peroxidation pathways involve enzymes such as arachidonic acid 15-lipoxygenase (ALOX15), which catalyse the oxidation of unsaturated fatty acids, leading to lipid peroxidation and cell death [49]. To elucidate the involvement of lncRNA-CFTBS in ferroptosis, we analysed the expression levels of crucial proteins associated with this process. The results revealed that OE-Lnc-CFTBS reduced FTH1 expression and increased ALOX15 expression, whereas SLC7A11 and GPX4 levels remained unchanged. These findings suggest that lncRNA-CFTBS promotes ferroptosis through pathways involving iron and lipid peroxidation rather than through the classical cystine/GSH/GPX4 axis (Figure 2(G)). In summary, these results establish that lncRNA-CFTBS does not affect autophagy and necrosis pathways but is crucial in driving ferroptosis through effects on peroxidation, iron metabolism, and ROS levels rather than the GPX4 pathway.

(A) CFU results showing BCG survival in THP-1 cells and OE-LncRNA cells treated with different pathway inhibitors. (B-H) Analysis of cellular changes following BCG infection in lncRNA-CFTBS-knockdown and -overexpressing cell lines: analysis of intracellular Fe^2+^ levels (B), ROS levels (C), mean fluorescence intensity quantification (D), MDA levels (E), TEM images illustrating ultrastructural changes (F; scale bar: 500 nm), and western blot analysis (G). The data are presented as means ± SDs, with significance levels indicated by * (*p* < 0.05), ** (*p* < 0.01), *** (*p* < 0.005), and ns (not significant).

### LncRNA-CFTBS regulates ferroptosis via the miR-515-5p/miR-519e-5p-SAT1 axis during M.tb infection

**Figure 3.**
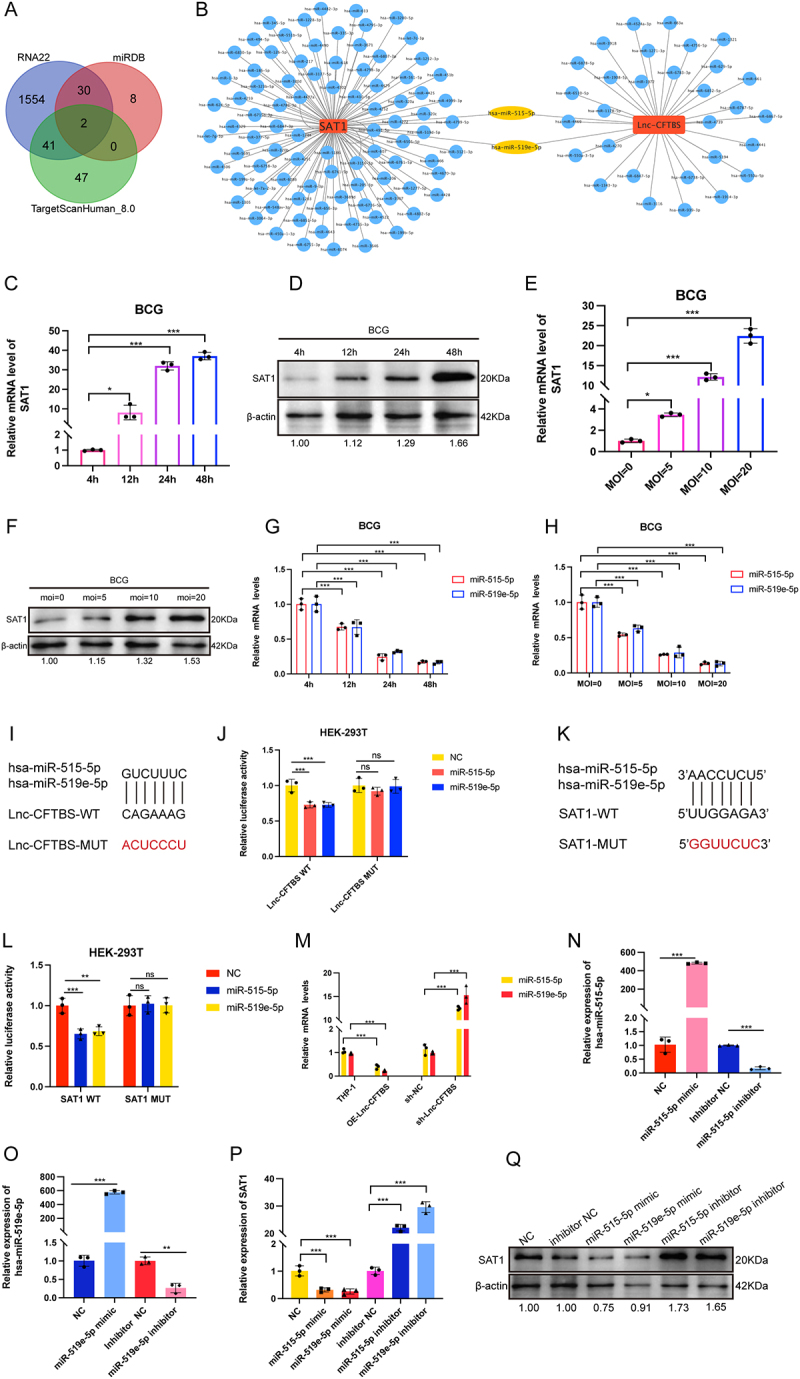
miR-515-5p/miR-519e-5p participation in the interaction between lncRNA-CFTBS and SAT1.

We found that elevated levels of lncRNA-CFTBS during BCG exposure enhance its persistence in macrophages by regulating the ferroptosis pathway. We further investigated the mechanism through which lncRNA-CFTBS exerts its effects. Given that most cytoplasm-localized lncRNAs function by acting as molecular sponges for miRNAs, we hypothesized that lncRNA-CFTBS plays a similar role. Using RNA22 (cm.jefferson.edu/rna22) and miRDB (mirbase.org), we subsequently screened for potential miRNAs that interact with lncRNA-CFTBS. The results are provided in supplementary Excel files (Table S2). Additionally, from our previous transcriptomic data, we filtered genes with significantly upregulated expression during M.tb infection, focusing on genes involved in iron metabolism and lipid metabolism pathways associated with ferroptosis, excluding the GPX4 pathway. SAT1 was identified as a key gene in these pathways and is known for its role in polyamine catabolism, which can influence iron metabolism and lipid peroxidation, both of which are crucial for ferroptosis. We identified miRNAs that potentially regulate SAT1 via a negative regulatory model. Cross-referencing the datasets revealed that only miR-515-5p and miR-519e-5p were present in the overlapping regions (Figure 3(A,B)). We hypothesized that lncRNA-CFTBS modulates ferroptosis by regulating SAT1 through these miRNAs, thereby playing a functional role in this pathway. We examined SAT1 expression in cells exposed to H37Rv at various MOIs and time points and detected significant upregulation (Figure S3A and S3B), which was consistent with our sequencing results. We then examined SAT1 expression in cells at different intervals after BCG infection, revealing significant upregulation (Figure 3(C,D)). Additionally, SAT1 mRNA and protein expression were consistently upregulated in THP-1 cells infected with BCG at various MOIs (Figure 3(E,F)). In addition, we analysed miR-515-5p/miR-519e-5p expression levels at various time points post-H37Rv infection and detected significant downregulation of these miRNAs (Figure S3C), which was consistent with the results at different MOIs (Figure S3D). Furthermore, we examined the expression levels of these miRNAs at various time points post-BCG infection (Figure 3(G)) and at different MOIs (Figure 3(H)). The results indicated significant downregulation of miR-515-5p/miR-519e-5p following infection.

To validate the interactions between lncRNA-CFTBS and miR-515-5p/miR-519e-5p as well as between SAT1 and miR-515-5p/miR-519e-5p, we constructed dual-luciferase reporter vectors for lncRNA-CFTBS-WT and lncRNA-CFTBS-MUT to confirm the regulatory relationship (Figure 3(I)). The dual-luciferase assay results (Figure 3(J)) indicated that cotransfection of lncRNA-CFTBS-WT with these miRNAs mimics significantly reduced luciferase activity compared to the negative control group. In contrast, luciferase activity remained unchanged in the lncRNA-CFTBS-MUT group, confirming that lncRNA-CFTBS directly interacts with these miRNAs. In addition, to establish that SAT1 is regulated by miR-515-5p/miR-519e-5p, we constructed SAT1-WT and SAT1-MUT vectors (Figure 3(K)). The results confirmed that cotransfection of SAT1-WT with these miRNAs mimics reduced luciferase activity, whereas the SAT1-MUT group showed no change (Figure 3(L)), indicating that these miRNAs directly target SAT1. Additionally, we further explored the modulatory impact of lncRNA-CFTBS on miR-515-5p/miR-519e-5p expression. RT‒qPCR analysis of RNA samples from OE-Lnc-CFTBS and sh-Lnc-CFTBS cells revealed an inverse relationship between lncRNA-CFTBS and the expression levels of these miRNAs (Figure 3(M)). This observation was further confirmed by increased miRNA expression upon si-Lnc-CFTBS transfection in THP-1 cells (Figure S3E). To investigate the function of miR-515-5p and miR-519e-5p, initially, the transfection efficiency of these miRNAs mimics and inhibitors was verified by RT‒qPCR, which revealed significant changes suitable for subsequent experiments (Figure 3(N,O)). Additional RT-qPCR showed an inverse correlation between miR-515-5p/miR-519e-5p and SAT1 levels (Figure 3(P)), confirmed by western blot at the protein level (Figure 3(Q)). The successful generation of cell lines overexpressing or silencing miR-515-5p/miR-519e-5p (OE-miR-515-5p/OE-miR-519e-5p and sh-miR-515-5p/sh-miR-519e-5p) was validated, and subsequent experiments revealed a negative correlation between these miRNAs and SAT1 expression (Figures S3F, S3G, and S3H). In summary, we identified the regulatory relationships among lncRNA-CFTBS, miR-515-5p/miR-519e-5p, and SAT1 during the process of BCG infection.

(A) Venn diagram showing potential miRNAs that interact with lncRNA-CFTBS and regulate SAT1 identified. (B) Network analysis illustrating the lncRNA‒miRNA-SAT1 regulatory network. (C-F) SAT1 mRNA expression and protein levels in THP-1 cells at various time points post-BCG infection and at different MOIs. (G-H) RT‒qPCR analysis of miR-515-5p and miR-519e-5p expression levels in THP-1 cells at various time points and different MOIs post-BCG infection. (I) Schematic of the lncRNA-CFTBS-WT and lncRNA-CFTBS-MUT constructs. (J) Dual-luciferase reporter assay results for HEK-293T cells cotransfected with lncRNA-CFTBS-WT or lncRNA- CFTBS-MUT and these miRNAs mimics or NC. (K) Schematic of SAT1-WT and SAT1-MUT constructs. (L) Dual-luciferase reporter assay results for HEK-293T cells cotransfected with SAT1-WT or SAT1-MUT and these miRNAs mimics. (M-O) RT-qPCR analysis of miR-515-5p and miR-519e-5p expression levels in cell lines with lncRNA-CFTBS overexpression or silencing, as well as in THP-1 cells transfected with respective mimics, inhibitors. (P‒Q) SAT1 mRNA and protein levels in THP-1 cells transfected with miR-515-5p/miR-519e-5p mimics and inhibitors. The data are presented as means ± SDs, with significance levels indicated by * (*p* < 0.05), ** (*p* < 0.01), *** (*p* < 0.005), and ns (not significant).

### miR-515-5p and miR-519e-5p regulate the intracellular survival of BCG through the ferroptosis pathway

**Figure 4.**
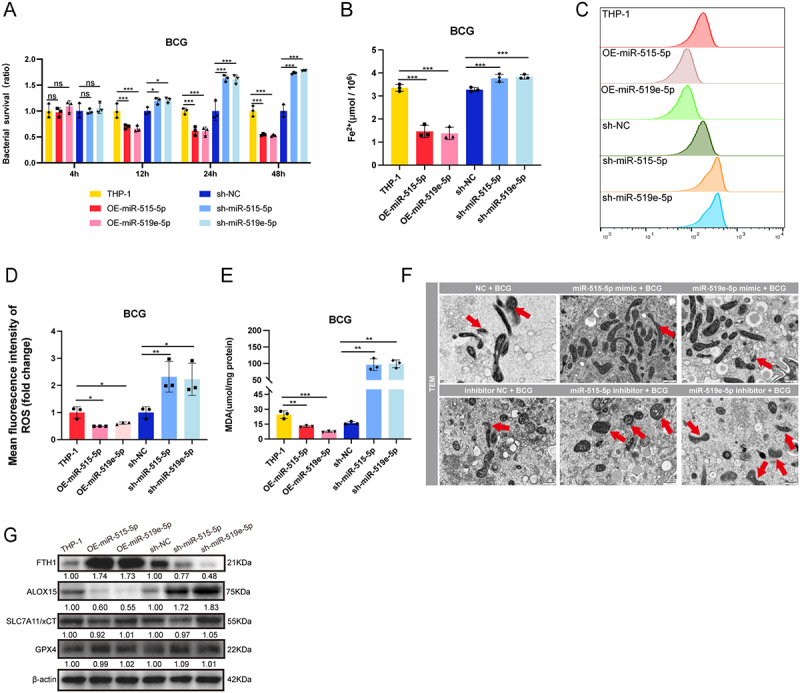
miR-515-5p/miR-519e-5p modulate ferroptosis and regulate the intracellular survival of BCG.

Previous studies have demonstrated that lncRNA-CFTBS influences BCG survival within cells through the ferroptosis pathway and competitively binds miR-515-5p/miR-519e-5p, regulating the downstream gene SAT1. Our investigation into the function of these miRNAs via CFU assays post-BCG infection in OE-miR-515-5p/miR-519e-5p and sh-miR-515-5p/miR-519e-5p cells revealed that these miRNAs overexpression reduced BCG survival, whereas silencing these miRNAs promoted it (Figure 4(A)). BCG survival was similarly influenced by transfection with miRNA mimics and inhibitors (Figure S4A).

To verify whether miR-515-5p/miR-519e-5p regulate the ferroptosis pathway, we measured relevant markers associated with this pathway. The levels of intracellular Fe^2+^ were significantly decreased by the overexpression of these miRNAs and increased by the silencing of these miRNAs (Figure 4(B)). Their role in regulating ROS levels was further examined via flow cytometry. Flow cytometry confirmed that the silencing of these miRNAs increased the fluorescence intensity of ROS, whereas the overexpression of these miRNAs reduced it (Figure 4(C,D)). MDA levels were assessed to determine the impact on ferroptosis, and the results revealed that silencing these miRNAs significantly increased MDA levels, whereas overexpressing these miRNAs reduced them (Figure 4(E)). TEM analysis provided insight into mitochondrial changes, revealing that the inhibition of these miRNAs led to smaller mitochondria with disrupted cristae (Figure 4(F)). Counting showed that the proportion of damaged mitochondria was 34.48% in the NC BCG group, 11.90% in the miR-515-5p mimic BCG group, 9.09% in the miR-519e-5p mimic BCG group, 22.58% in the inhibitor NC BCG group, 71.43% in the miR-515-5p inhibitor BCG group, and 70.00% in the miR-519e-5p inhibitor BCG group. Further investigation of ferroptosis-related proteins via western blotting revealed that silencing these miRNAs reduced FTH1 expression and increased ALOX15 without significantly altering GPX4 and SLC7A11 levels, indicating that these miRNAs play roles in modulating key proteins involved in ferroptosis (Figure 4(G)). These findings underscore the regulatory influence of miR-515-5p/miR-519e-5p on BCG survival and the ferroptosis pathway.

(A) CFU assay results showing the bacterial survival ratios of BCG in cell. (B-H) Analysis of changes in OE-miR-515-5p/OE-miR-519e-5p and sh-miR-515-5p/sh-miR-519e-5p cells post-BCG infection: intracellular Fe^2+^ levels (B), ROS detection: flow cytometry histograms displaying ROS levels (C), quantification of the mean fluorescence intensity (D), quantification of MDA levels (E), TEM images showing the mitochondrial ultrastructure (F; Scale bar: 500 nm), and analysis of ferroptosis-related proteins (G). The data are presented as means ± SDs, with significance levels indicated by * (*p* < 0.05), ** (*p* < 0.01), *** (*p* < 0.005), and ns (not significant).

### SAT1 enhances the intracellular survival of BCG

**Figure 5.**
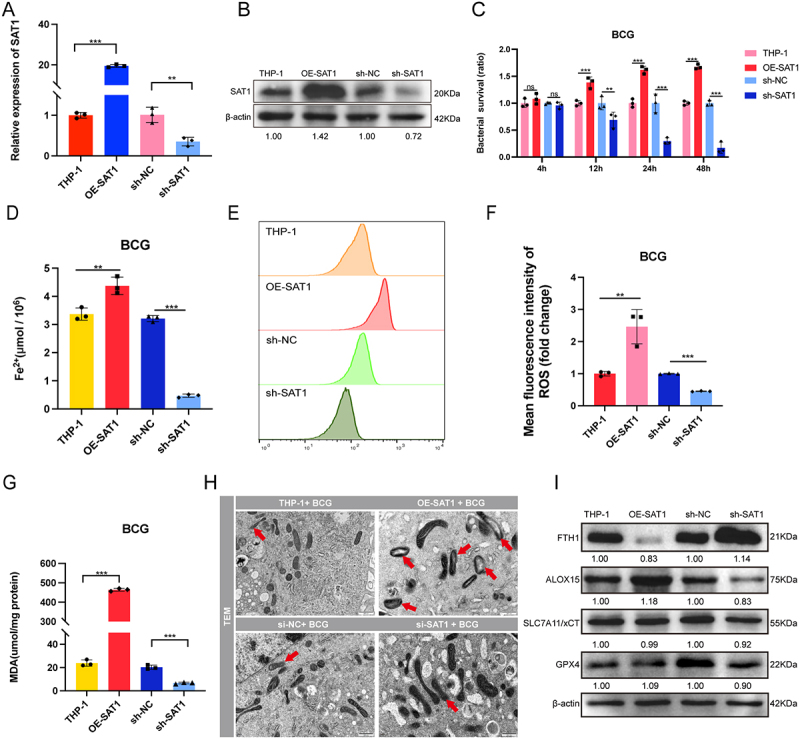
Role of SAT1 in promoting ferroptosis and enhancing BCG survival.

In our previous work, we validated the roles of lncRNA-CFTBS and miR-515-5p/miR-519e-5p within the lncRNA-CFTBS-miR-515-5p/miR-519e-5p-SAT1 regulatory axis. We subsequently conducted functional studies on SAT1 during M.tb infection. We constructed cell lines overexpressing SAT1 (OE-SAT1) and with SAT1 silencing (sh-SAT1), and assessments of these cell lines confirmed successful overexpression and silencing of SAT1 (Figure 5(A,B)). To assess the impact of SAT1 on BCG survival, we performed CFU assays using OE-SAT1 and sh-SAT1 cells post-BCG infection. The data revealed that OE-SAT1 promoted BCG survival, whereas sh-SAT1 inhibited it, with sh-SAT1 suppressing BCG survival by 80% at 48 hours post infection (Figure 5(C)). Additionally, we performed transient SAT1 siRNA transfections. In THP-1 cells, siRNA-mediated SAT1 knockdown revealed that si-SAT1–2 was the most effective, and therefore, it was selected for subsequent experiments (Figure S5A and S5B). CFU assays post-BCG infection in si-SAT1-transfected cells revealed that si-SAT1 significantly inhibited BCG survival (Figure S5C). Similarly, CFU assays after H37Rv infection in si-SAT1-transfected cells revealed a significant reduction in H37Rv survival (Figure S5D).

We infected OE-SAT1 and sh-SAT1 cells with BCG for subsequent experiments, including Fe^2+^ quantification, MDA measurements, ROS analysis, TEM imaging, and western blotting. Compared with that in THP-1 cells, the level of cellular Fe^2+^, which participates in the Fenton reaction and is positively correlated with ferroptosis, was significantly higher in OE-SAT1 cells and lower in sh-SAT1 cells compared to their respective controls (Figure 5(D)). To assess the impact of SAT1 on ROS levels post-BCG infection, we utilized flow cytometry. The analysis revealed that ROS mean fluorescence intensity was higher in OE-SAT1 cells than in in THP-1 cells but lower in sh-SAT1 cells than in shNC cells (Figure 5(E,F)). Furthermore, we examined the impact of SAT1 on MDA levels post-BCG infection. Our results demonstrated that MDA levels were markedly elevated in OE-SAT1 cells compared to THP-1 cells but lower in sh-SAT1 cells than in shNC cells (Figure 5(G)). TEM analysis revealed that, compared with the control, SAT1 overexpression caused mitochondrial membrane disruption, led to a reduction in and disordered cristae, and transformed mitochondria from typical elongated shapes to irregular forms (Figure 5(H)). Counting indicated that SAT1 overexpression increased the proportion of damaged mitochondria from 26.32% (THP-1 BCG) to 61.54%, while SAT1 knockdown reduced this proportion from 24.14% (siNC BCG) to 13.33%. Western blot analysis was subsequently performed to assess the expression of key proteins. Our findings revealed that SAT1 overexpression led to decreased FTH1 expression and increased ALOX15 expression, whereas the expression levels of SLC7A11 and GPX4 remained unchanged compared with those in THP-1 cells (Figure 5(I)). Our findings align with those of previous studies, confirming the role of SAT1 in regulating the lipid peroxidation pathway during M.tb infection [9,16]. The increased ferroptosis induced by the upregulation of SAT1 expression further enhances the intracellular survival of the pathogen.

(A-B) SAT1 mRNA and protein levels in various cell lines in which SAT1 was overexpressed or silenced. (C) Bacterial survival ratio of BCG in THP-1, OE-SAT1, sh-NC, and sh-SAT1 cells. (D-I) Analysis of changes in SAT1-overexpressing and SAT1-knockdown cells after BCG infection: intracellular Fe^2+^ levels (D), ROS detection (E) and mean fluorescence intensity (F), measurement of MDA levels (G), TEM images of cell lines (H; scale bars: 500 nm), and protein level analysis (I). The data are presented as means ± SDs, with significance levels indicated by * (*p* < 0.05), ** (*p* < 0.01), *** (*p* < 0.005), and ns (not significant).

### Regulation of ferroptosis by miR-515-5p/miR-519e-5p through SAT1

To investigate the role of miR-515-5p and miR-519e-5p in modulating ferroptosis via the SAT1 pathway during BCG infection, we performed several assays at various time points and under various conditions. Cells were cotransfected with these miRNAs mimics and SAT1, followed by BCG infection. We then assessed intracellular Fe^2+^ levels, ultrastructure via TEM, MDA content, and ROS levels.

**Figure 6.**
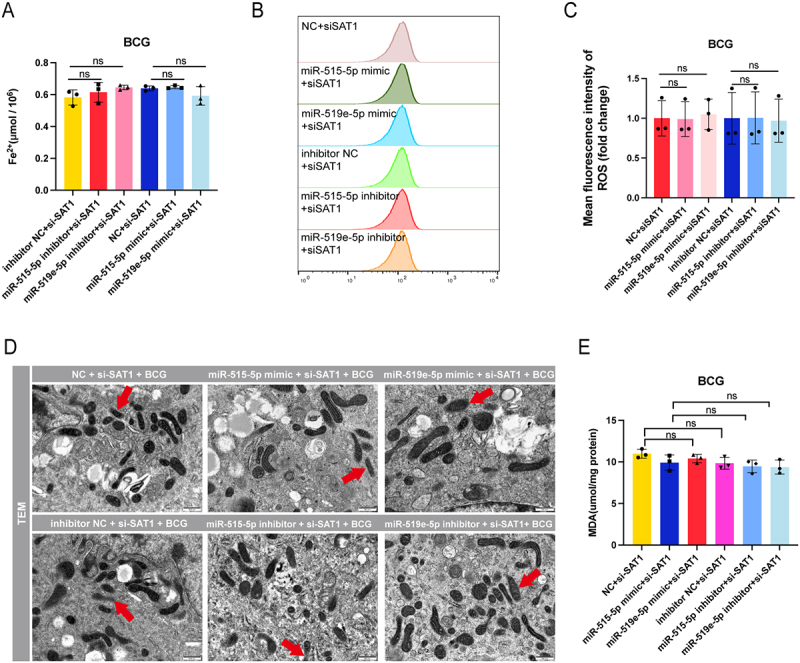
miR-515-5p/miR-519e-5p modulate ferroptosis via SAT1.

The findings suggested that cotransfection with si-SAT1 and these miRNAs mimics and inhibitors did not significantly alter intracellular Fe^2+^ levels in BCG-infected cells (Figure 6(A)). Additionally, ROS levels were assessed, and flow cytometry revealed no significant difference when si-SAT1 was cotransfected with these miRNAs mimics or inhibitors (Figure 6(B,C)). TEM images revealed that cotransfection of si-SAT1 with these miRNAs mimics or inhibitors resulted in no significant differences in mitochondrial morphology compared with that observed in the control group (Figure 6(D)). Counting showed that the proportion of damaged mitochondria remained comparable across all groups, including NC si-SAT1 (21.79%), miR-515-5p mimic si-SAT1 (21.43%), miR-519e-5p mimic si-SAT1 (22.50%), inhibitor NC si-SAT1 (22.22%), miR-515-5p inhibitor si-SAT1 (21.43%), and miR-519e-5p inhibitor si-SAT1 (20.51%). MDA content analysis revealed that cotransfection of si-SAT1 with these miRNAs mimics or inhibitors had no significant effect, indicating that these miRNAs promote lipid peroxidation through SAT1 (Figure 6(E)). These findings collectively suggest that miR-515-5p/miR-519e-5p modulate ferroptosis by regulating lipid peroxidation and ROS levels through the SAT1 pathway.

The following were assessed after cotransfection with si-SAT1 and miR-515-5p/miR-519e-5p mimics and inhibitors in BCG-infected cells: intracellular Fe^2+^ levels (A), ROS detection (B), mean fluorescence intensity (C), TEM images displaying mitochondrial changes (D), and MDA content (E). The data are presented as means ± SDs, with significance levels indicated by * (*p* < 0.05), ** (*p* < 0.01), *** (*p* < 0.005), and ns (not significant).

### LncRNA-CFTBS regulates ferroptosis via miR-515-5p/miR-519e-5p

**Figure 7.**
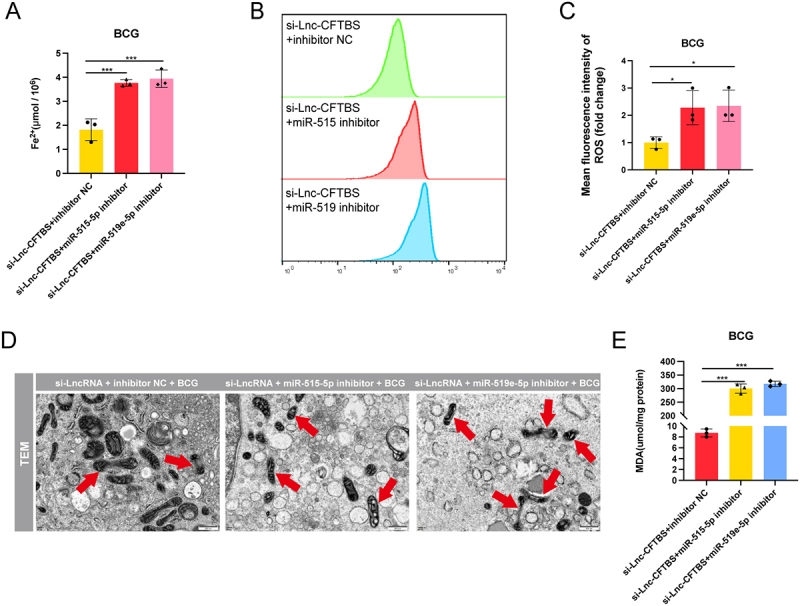
LncRNA-CFTBS regulates ferroptosis via miR-515-5p/miR-519e-5p.

Our previous findings highlighted the role of miR-515-5p/miR-519e-5p in regulating ferroptosis through SAT1. We further explored how lncRNA-CFTBS might influence this process through these miRNAs. Fe^2+^ assays (Figure 7(A)) revealed that these miRNAs inhibitors increased Fe^2+^ levels when cotransfected with si-lncRNA-CFTBS. The flow cytometry data (Figure 7(B,C)) indicated that the ROS levels increased with the cotransfection of si-lncRNA-CFTBS and miR-515-5p/miR-519e-5p inhibitors. TEM revealed more severe mitochondrial damage in cells cotransfected with si-lncRNA-CFTBS and miR-515-5p/miR-519e-5p inhibitors, characterized by mitochondrial shrinkage, disrupted cristae, and overall disorganization (Figure 7(D)). Counting showed that the proportion of damaged mitochondria increased from 25.00% in the inhibitor NC group to 72.22% and 81.48% in the miR-515-5p and miR-519e-5p inhibitor groups, respectively. MDA assays revealed that cotransfection of si-lncRNA-CFTBS with these miRNAs inhibitors significantly increased MDA levels, indicating enhanced lipid peroxidation (Figure 7(E)). Collectively, these findings indicate that lncRNA-CFTBS influences ferroptosis through the modulation of miR-515-5p/miR-519e-5p, affecting critical ferroptosis markers such as MDA, Fe^2+^, ROS, and mitochondrial integrity.

The following were evaluated in THP-1 cells transfected with si-lncRNA-CFTBS and miR-515-5p/miR-519e-5p inhibitors post-BCG infection: intracellular Fe^2+^ levels (A), flow cytometry analysis and MFI of ROS (B and C), TEM images depicting mitochondrial morphology (D; scale bars: 500 nm), and MDA levels (E). The data are presented as means ± SDs, with significance levels indicated by * (*p* < 0.05), ** (*p* < 0.01), *** (*p* < 0.005), and ns (not significant).

## Discussion

**Figure 8.**
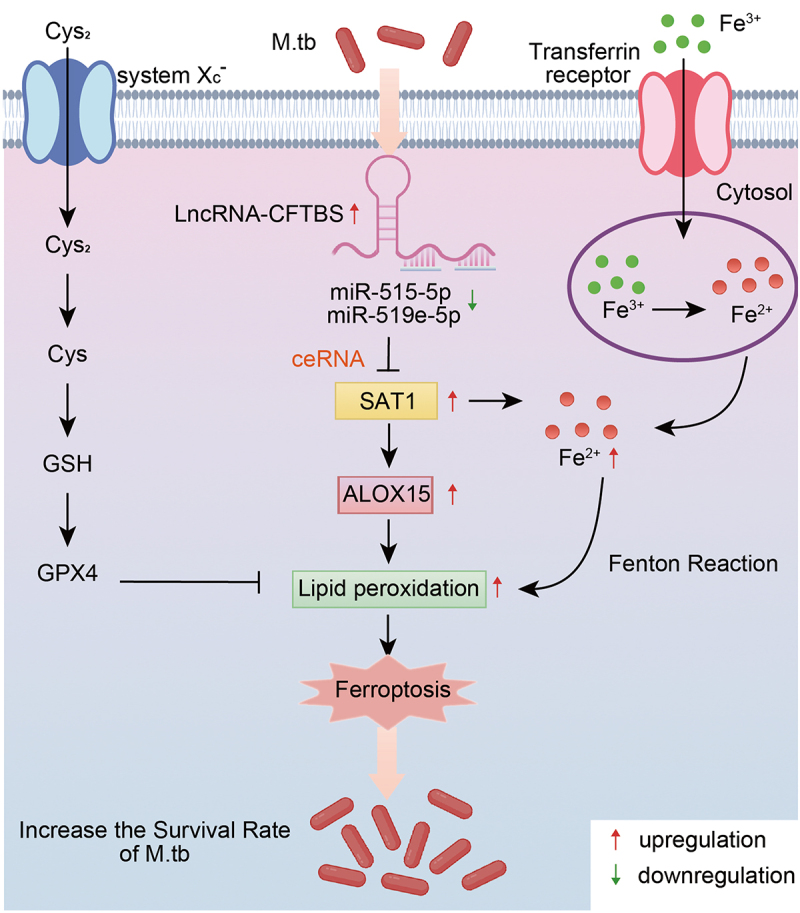
A schematic model of the role of lncRNA-CFTBS in M.tb infection.

In this study, we investigated the role of lncRNA-CFTBS during M.tb exposure, with a specific focus on its interactions with miR-515-5p and miR-519e-5p and the subsequent downstream effects on SAT1 expression and ferroptosis pathways. Our study revealed that lncRNA-CFTBS competitively binds miR-515-5p and miR-519e-5p, regulating their target gene SAT1 and subsequently affecting ferroptosis pathways. This regulation significantly influences M.tb survival in cells (Figure 8).

Many studies have elucidated the pivotal functions of lncRNAs in the pathogenesis of M.tb. LncRNAs regulate gene expression by functioning as miRNA sponges, competing endogenous RNAs (ceRNAs), decoys, or scaffolds [28,50–53]. Such interactions are central to various cellular processes, including those involved in disease pathogenesis [54–56]. Eduardo et al. identified lncRNAs as crucial regulators of immune response-related genes in TB [57]. Analyses of lncRNA expression patterns suggest that lncRNAs may significantly influence TB pathophysiology and progression [58]. The lncRNA NR_003508 plays a role in inhibiting programmed necrosis within a specific range and controlling the spread of M.tb [59]. The lncRNA DANCR induces autophagy and restrains M.tb survival within macrophages [60]. LncRNA-EST12 inhibits anti-M.tb innate immunity via FUBP3 [61]. LncRNA-CGB expression is downregulated during TB infection due to dysbiosis of the gut microbiota. Increasing the expression of lncRNA-CGB can promote anti-TB immunity [62]. Our group’s previous sequencing results revealed that lncRNA-CFTBS expression was significantly upregulated following M.tb infection, suggesting its potential role in the host‒pathogen interaction during TB. This discovery led us to explore the role of lncRNA-CFTBS in M.tb pathogenesis more deeply. Similarly, by integrating our previous transcriptome sequencing data with miRNA predictions, we determined that lncRNA-CFTBS competitively binds miR-515-5p and miR-519e-5p, thereby regulating their availability and consequently modulating the expression of their target gene, SAT1.

SAT1 is an enzyme involved in polyamine catabolism and has been increasingly recognized for its role in ferroptosis [9]. The understanding of ferroptosis has significantly expanded in recent years, revealing its critical roles in various pathological conditions, including neurodegeneration, cancer, and infection-related diseases [63–65]. The upregulation of SAT1 expression has been shown to promote ferroptosis by enhancing lipid peroxidation [66]. Recent studies have emphasized the interaction between SAT1 and ferroptosis in various disease contexts. For example, the overexpression of SAT1 has been linked to increased susceptibility to ferroptosis in Alzheimer’s disease, suggesting potential therapeutic avenues for Alzheimer’s disease treatment by induction of ferroptotic cell death [67]. In the context of RNF113A deficiency, increased SAT1 expression leads to increased ferroptosis, aiding in cell death and overcoming chemotherapy resistance [68]. SAT1 reduces the sensitivity of lung adenocarcinoma cells to chemotherapy by regulating ferroptosis [69]. While numerous studies have shown that SAT1 is a regulator of ferroptosis in various diseases, the mechanisms by which M.tb influences SAT1 to impact ferroptosis remain unexplored. Recent research has indicated that SAT1 expression is significantly elevated in clinical blood samples from tuberculosis patients [70]. In our study, we provide evidence that SAT1 induces ferroptosis in the context of M.tb infection, which aligns with earlier findings [9,16,71]. We found that lncRNA-CFTBS expression levels increased, sequestering miR-515-5p and miR-519e-5p, which resulted in the modulation of SAT1 during M.tb infection. Our data indicate that this interaction resulted in the upregulation of SAT1 expression, which promoted ferroptosis, thereby creating an intracellular environment conducive to M.tb survival. This highlights a pathway by which lncRNA-CFTBS can modulate host‒pathogen interactions through ferroptosis. The overexpression of lncRNA-CFTBS led to increased levels of MDA, Fe^2+^, and ROS. At the protein level, lncRNA-CFTBS overexpression significantly altered FTH1 and ALOX15 levels but had no notable effect on GPX4 or SLC7A11. These results suggest that lncRNA-CFTBS induces ferroptosis via lipid peroxidation pathways rather than the classical cystine/GSH/GPX4 axis.

Although some studies have indicated that ferroptosis contributes to M.tb pathogenesis, the specific role of lncRNAs in this process remains underexplored. Our research addresses this gap, demonstrating that lncRNA-CFTBS is a critical player in that regulatory axis. This study provides a deeper understanding of the complex regulatory networks involving lncRNAs, miRNAs, and ferroptosis in M.tb infection. By elucidating the role of the lncRNA-CFTBS/miR-515-5p/miR-519e-5p/SAT1 axis, we highlight a potential therapeutic target for TB treatment. Modulating this pathway could reduce bacterial survival, offering new strategies for combating this persistent disease. Despite these promising findings, our study has several limitations. Our study focused primarily on the molecular interactions and regulatory pathways involving lncRNA-CFTBS, miR-515-5p/miR-519e-5p, and SAT1. While we have demonstrated the involvement of these molecules in ferroptosis, other regulatory elements and signalling pathways might also play a role. A more comprehensive analysis involving additional molecular players could provide a deeper understanding of the ferroptosis regulatory network during M.tb infection.

## Conclusion

This research highlights the critical role of lncRNA-CFTBS in modulating ferroptosis during M.tb infection. We found that this regulation involves lipid metabolism and iron metabolism pathways rather than the classical cystine/GSH/GPX4 pathway. These results indicate that lncRNA-CFTBS regulates the intracellular environment to promote or inhibit M.tb survival by influencing ferroptosis, providing new insights into M.tb infection and identifying potential therapeutic targets for tuberculosis.

## Supplementary Material

Document S1.docx

## Data Availability

The transcriptional profiling data are available at the GEO database (GEO: GSE184660). The data that support the findings of this study are openly available in https://doi.org/10.6084/m9.figshare.28234085
