# Supplementary material for: LncRNA-CFTBS enhances Mycobacterium tuberculosis survival in macrophages by modulating ferroptosis through the miR-515-5p/miR-519e-5p/SAT1 axis
Source: Virulence. 2025 Oct 11;16(1):2545563. doi: 10.1080/21505594.2025.2545563 (PMC12520127; doi:10.1080/21505594.2025.2545563)
Supplement: Document S1.docx [file KVIR_A_2545563_SM3876.docx]

**
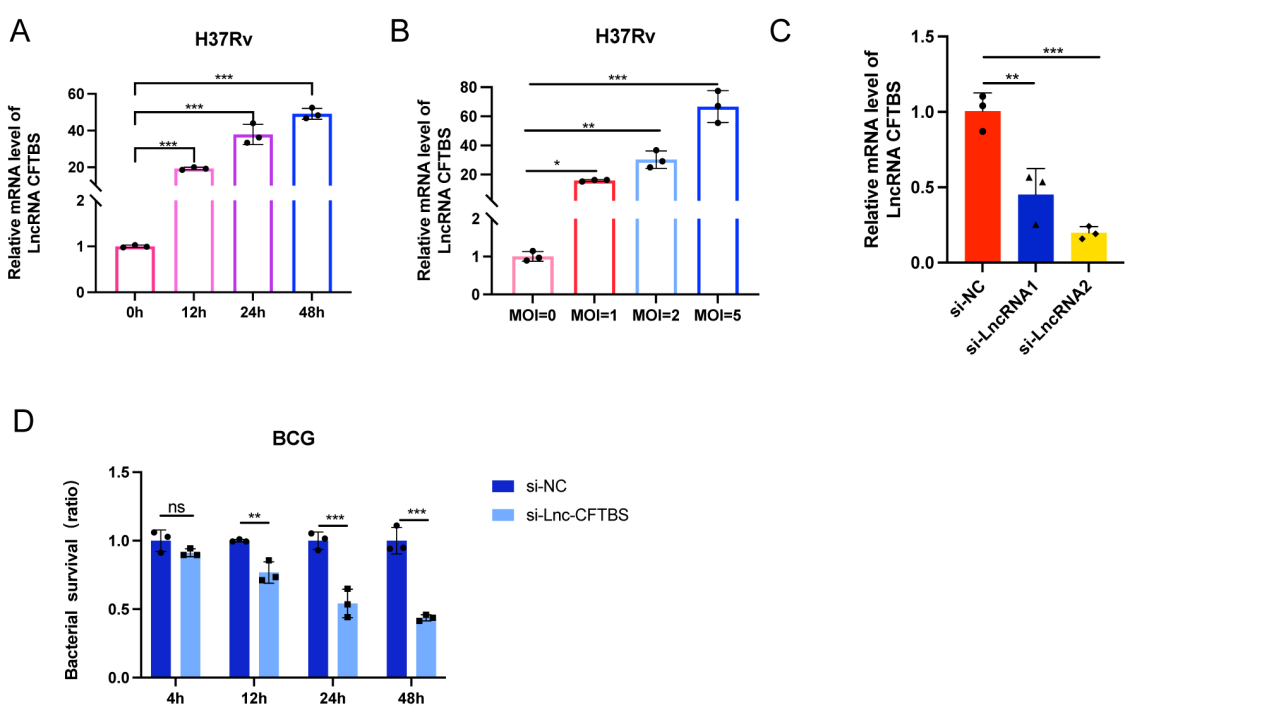
**

**Supplementary Figure S1**

(A-B) LncRNA-CFTBS expression in cells exposed to H37Rv at various time points and across different MOIs. (C) Validation of lncRNA-CFTBS silencing efficiency using siRNAs. (D) Impact of lncRNA-CFTBS silencing on BCG survival. The bacterial survival ratios were measured at 4 hours, 12 hours, 24 hours, and 48 hours post infection. The data are presented as means ± SDs. Statistical significance: ns =not significant, **p<0.01, and ***p<0.005.


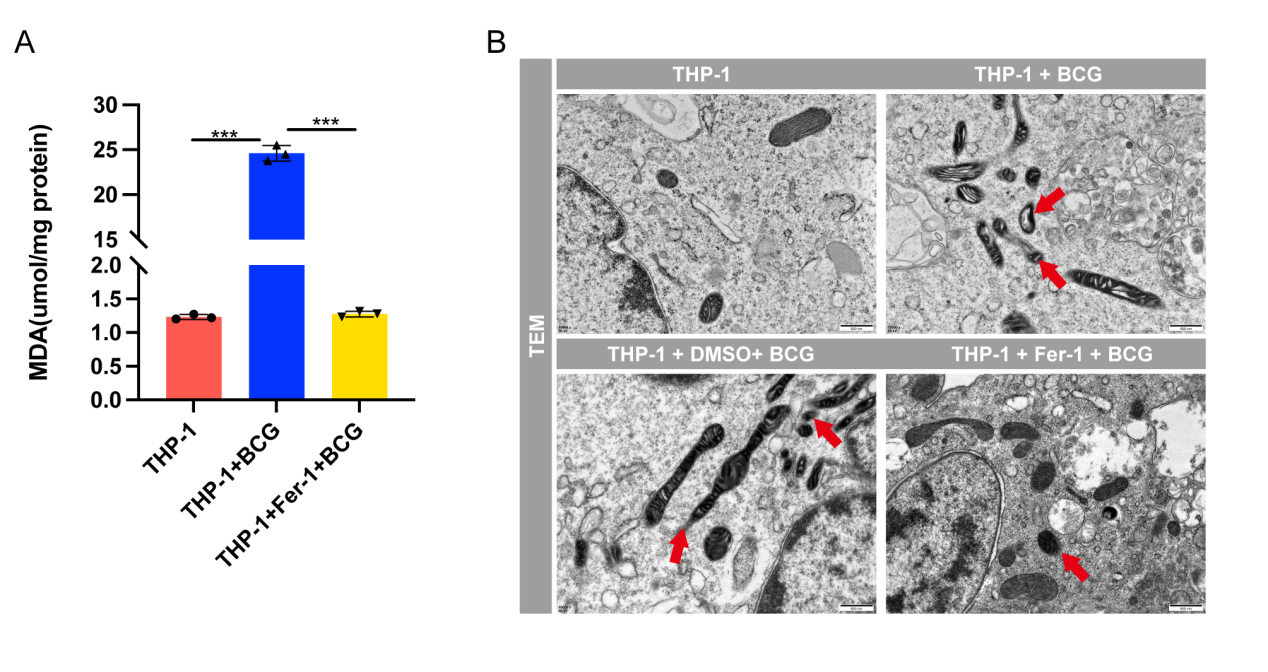


**Supplementary Figure S2**

(A) MDA levels in THP-1 cells infected with BCG, with or without the ferroptosis inhibitor Fer-1. (B) TEM images showing the mitochondrial ultrastructure. Scale bars represent 500 nm. The data are presented as means ± SDs, with significance levels indicated by * (p < 0.05), ** (p < 0.01), *** (p < 0.005), and ns (not significant).

**
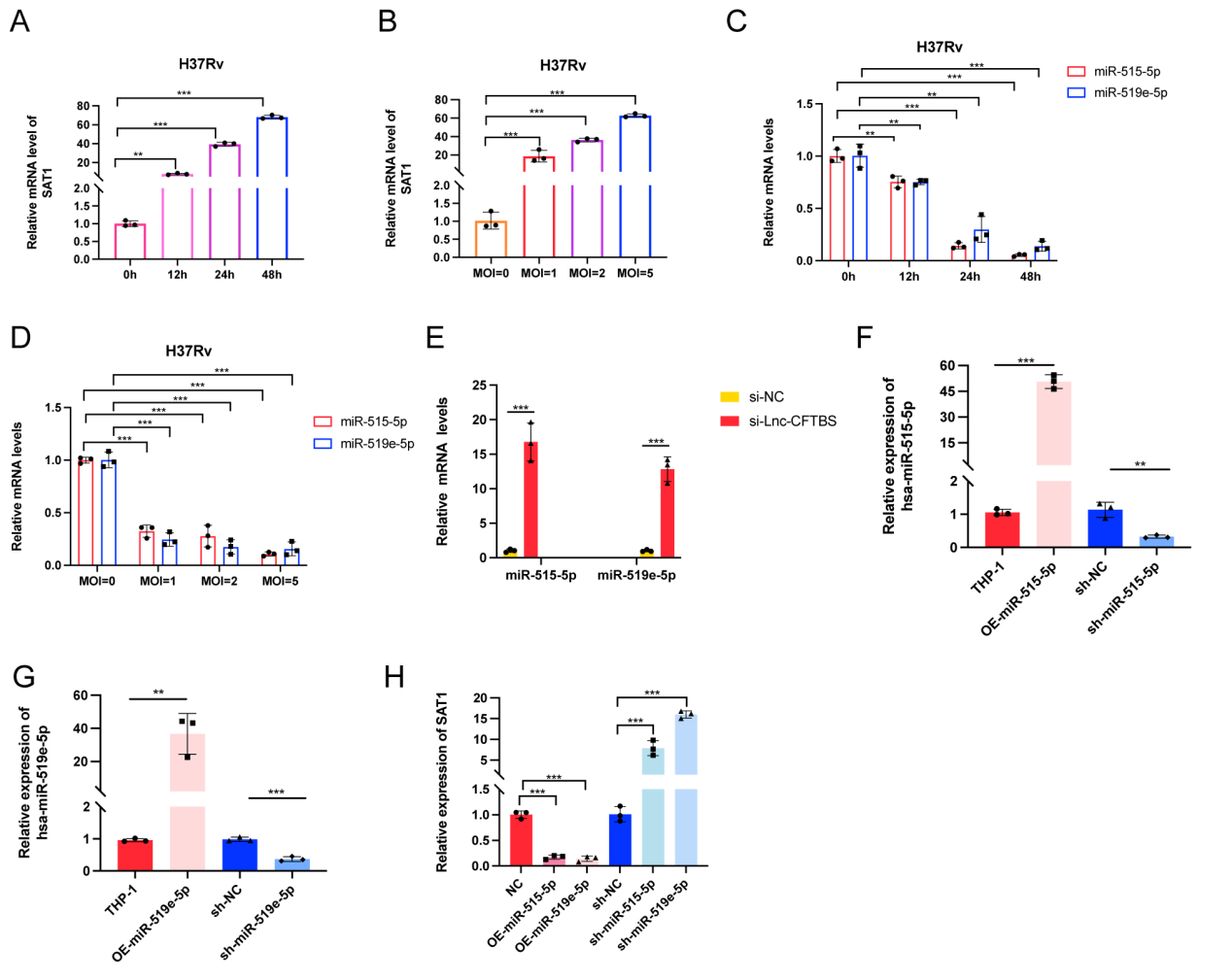
**

**Supplementary Figure S3**

(A-B) SAT1 mRNA at various time points post-H37Rv infection and at different MOIs (0, 1, 2, 5). (C-D) miR-515-5p and miR-519e-5p expression levels at various time points post-H37Rv infection and at different MOIs. (E) RT‒qPCR analysis of miR-515-5p and miR-519e-5p expression levels in the si-lncRNA-CFTBS or si-NC groups. (F-G) miR-515-5p expression in OE-miR-515-5p/sh-miR-515-5p cell lines and miR-519e-5p expression in OE-miR-519e-5p/sh-miR-519e-5p cell lines. (H) RT‒qPCR analysis of SAT1 expression in the OE-miR-515-5p/sh-miR-515-5p and OE-miR-519e-5p/sh-miR-519e-5p cell lines. The data are presented as means ± SDs, with significance levels indicated by * (p < 0.05), ** (p < 0.01), *** (p < 0.005), and ns (not significant).


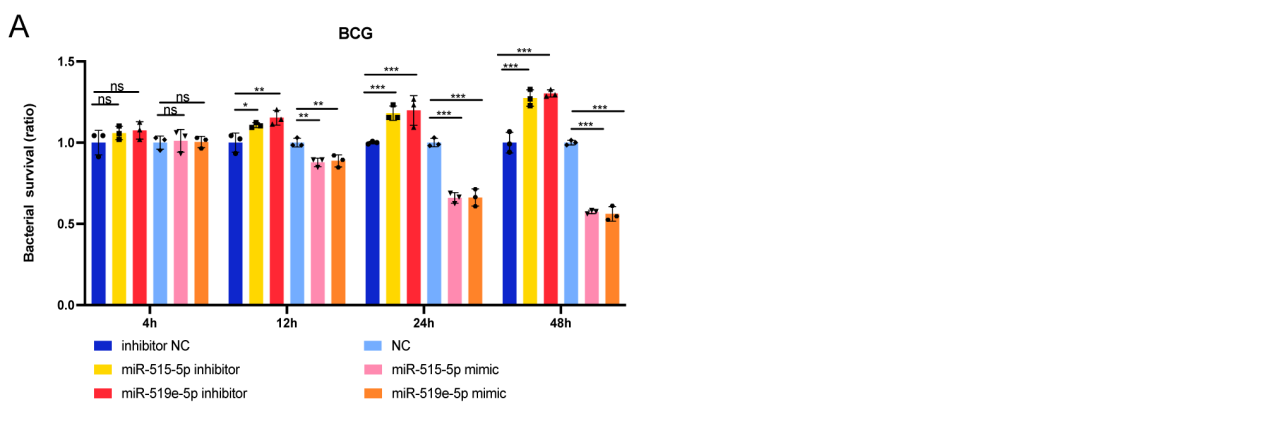


**Supplementary Figure S4**

(A) CFU assay results showing the bacterial survival ratios for BCG in the miR-515-5p/miR-519e-5p mimic and inhibitor groups. The data are presented as means ± SDs, with significance levels indicated by * (p < 0.05), ** (p < 0.01), *** (p < 0.005), and ns (not significant).


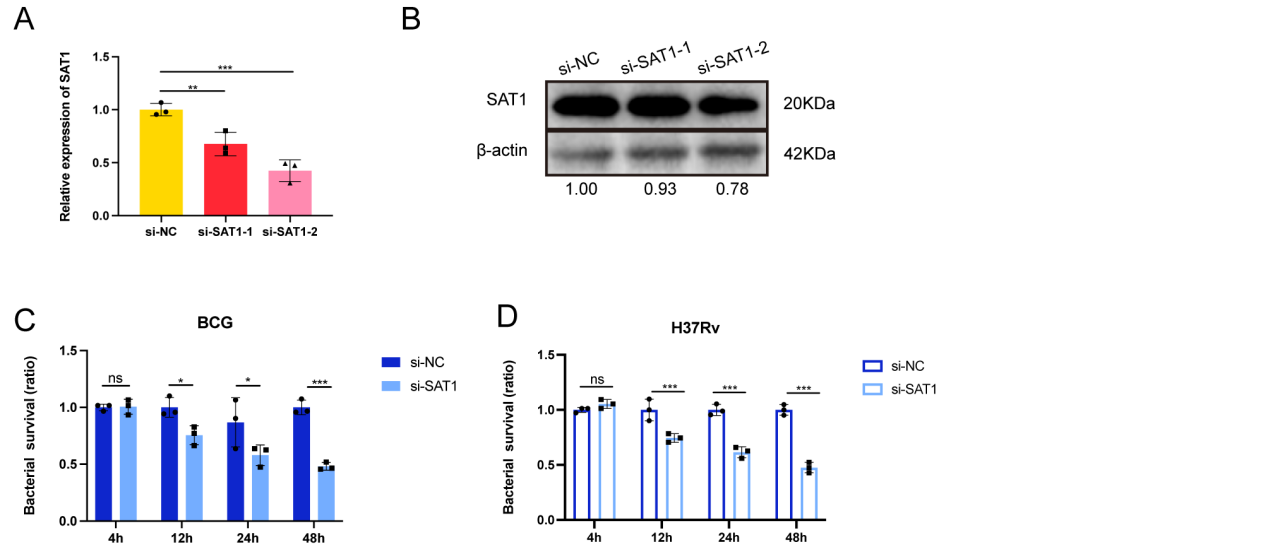


**Supplementary Figure S5**

(A-B) SAT1 mRNA and protein levels in the si-NC, si-SAT1-1, and si-SAT1-2 groups. (C-D) Bacterial survival ratios for BCG and H37Rv in si-NC- and si-SAT1-transfected cells at various time points post infection. The data are presented as means ± SDs, with significance levels indicated by * (p < 0.05), ** (p < 0.01), *** (p < 0.005), and ns (not significant).
